# Supplementary material for: miR-126 Decreases Proliferation and Mammosphere Formation of MCF-7 and Predicts Prognosis of ER+ Breast Cancer
Source: Diagnostics (Basel). 2022 Mar 18;12(3):745. doi: 10.3390/diagnostics12030745 (PMC8946945; doi:10.3390/diagnostics12030745)
Supplement: Supplementary file 1 [file diagnostics-12-00745-s001.zip › diagnostics-1433600-supplementary.pdf]

Supplementary Data

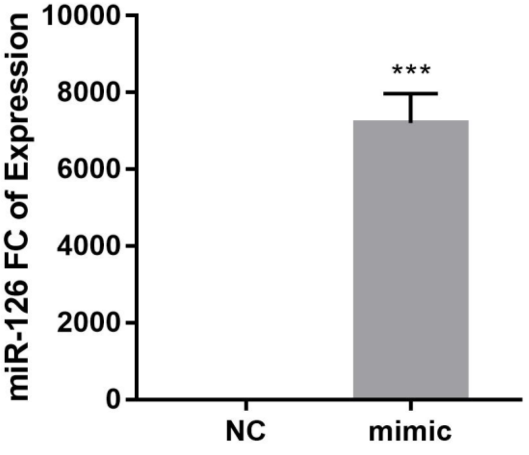

Figure S1. Transfection Efficiency of miR-126 mimic in MCF-7. RT-qPCR analysis of miR-126 levels as compared to NC in MCF-7 24hrs post-transfection with RNU6B as an endogenous control. Error bars represent SEM (n=3). *** denotes p<0.001.

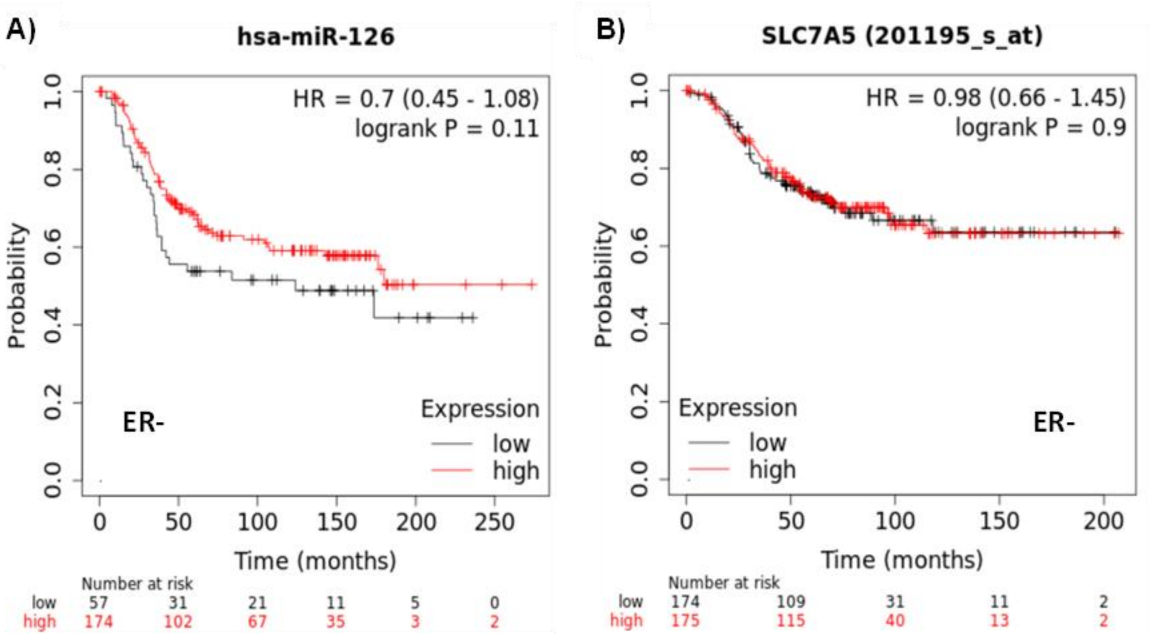

Figure S2. Correlation of the Expression of miR-126 or SLC7A5 with Overall Survival (OS) of ER- BC Patients. *In Silico* Kaplan-Meier Plot of (A): hsa-miR-126 expression with OS of 231 ER- BC patients, (B): SLC7A5 expression with OS of 349 ER- BC patients. HR: Hazard Ratio.

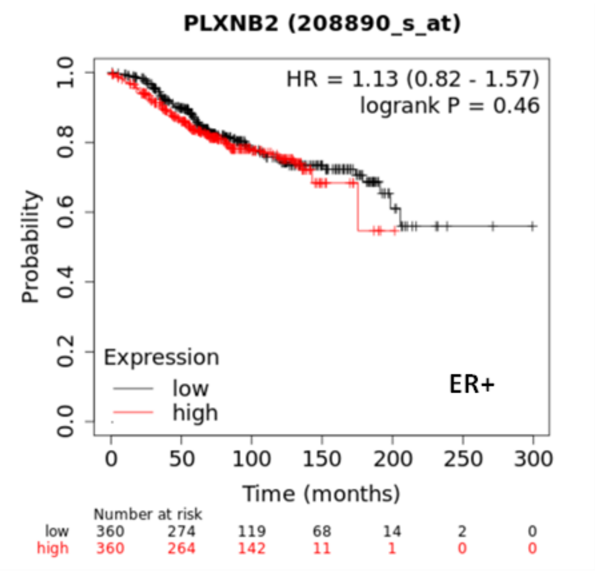

Figure S3. Correlation of the Expression of PLXNB2 with Overall Survival (OS) of ER+ BC Patients. *In Silico* Kaplan-Meier Plot of PLXNB2 expression with OS of 720 ER+ BC patients HR: Hazard Ratio.
